# Cross‐cultural prevalence of sleep quality and psychological distress in healthcare workers during COVID‐19 pandemic

**DOI:** 10.1002/brb3.2383

**Published:** 2021-10-17

**Authors:** Hamza Rafique Khan, Farzana Ashraf, Irfan Ullah, Muhammad Junaid Tahir, Asimina Dominari, Sheikh Shoib, Hamna Naeem, Gowry Reddy, Pramit Mukherjee, Ifrah Akram, Sudha Kamada, Roshni Riaz Memon, M. Muzzamil Yasin Khan, Sumit Raut, Mahmoud Mohamed Mohamed Shalaby, Rana Usman Anwar, Maheen Farooq, Krupa Ketankumar Soparia, Rodrigo Ramalho, Chung‐Ying Lin, Amir H. Pakpour

**Affiliations:** ^1^ Department of Medicine Quaid‐E‐Azam Medical College Bahawalpur Pakistan; ^2^ Department of Humanities COMSATS University Lahore Pakistan; ^3^ Kabir Medical College Gandhara University Peshawar Pakistan; ^4^ Naseer Teaching Hospital Peshawar Pakistan; ^5^ Ameer‐ud‐Din Medical College University of Health Sciences Lahore Pakistan; ^6^ Lahore General Hospital Lahore Pakistan; ^7^ Aristotle University of Thessaloniki School of Medicine Thessaloniki Greece; ^8^ Department of Psychiatry Jawahar Lal Nehru Memorial Hospital Srinagar India; ^9^ Karachi Medical and Dental College Karachi Pakistan; ^10^ Kasturba Medical College Manipal University Manipal India; ^11^ Department of Psychiatry Adichunchanagiri Institute of Medical Sciences (ACU) B.G. Nagara India; ^12^ NRI Institute of Medical Sciences Visakhapatnam India; ^13^ Ziauddin Medical College Karachi Pakistan; ^14^ Kathmandu Medical college Kathmandu Nepal; ^15^ Faculty of Medicine Ain Shams University Cairo Egypt; ^16^ Nishtar Medical University Multan Pakistan; ^17^ Jinnah Hospital Lahore Pakistan; ^18^ GMERS Medical College Gandhinagar India; ^19^ Department of Social and Community Health School of Population Health The University of Auckland Auckland New Zealand; ^20^ Institute of Allied Health Sciences College of Medicine National Cheng Kung University Tainan Taiwan; ^21^ Department of Nursing School of Health and Welfare Jönköping University Jönköping Sweden

**Keywords:** COVID‐19, healthcare workers, psychological distress, sleep disturbances, sleep quality

## Abstract

**Background:**

Poor quality sleep and emotional disturbances are expected in times of crisis. COVID‐19 has severely impacted healthcare worldwide and with that comes the concern about its effects on healthcare workers. The purpose of the present study was to assess sleep quality and psychological distress in healthcare workers during the COVID‐19 pandemic.

**Methods:**

The present work is a multi‐centric cross‐sectional study targeting healthcare workers from India, Pakistan, and Nepal. It used an online version of the Pittsburg Sleep Quality Index and the General Health Questionnaire, and data were analyzed using SPSS V.24.

**Results:**

A total of 1790 participants completed the questionnaire. Of the 1790 participants, 57% reported poor sleep quality, and 10% reported a high level of psychological distress. A cross‐cultural comparison found some differences between the different groups of participants. The details of the differences were further explored in the article.

**Conclusion:**

The present study highlights that a significant proportion of healthcare workers are affected by poor sleep quality and psychological distress during the COVID‐19 pandemic. It also emphasizes the imperative to provide them with psychosocial support to avoid potential short‐ and long‐term psychological consequences of these troubling times.

## Graphical abstract

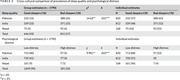

## 1 INTRODUCTION

Since the initial stages of the spreading of SARS‐CoV‐2, numerous reports have been made on the burden that the Coronavirus 2019 (COVID‐19) pandemic could impose on mental health (Ahorsu et al., 2020; Rajabimajd et al., 2021; Rajkumar, 2020; Webb, 2020; Xiang et al., 2020; Xiong et al., 2020). Healthcare workers were particularly exposed to this burden, as they were confronted with the duty of care while exposing themselves to the risk of contamination (Malik et al., 2021; Nguyen et al., 2020; Olashore et al., 2021). Many personal and environmental factors, such as an increased workload, physical exhaustion, often inadequate access to protective equipment, and making ethically difficult decisions may have dramatically affected healthcare workers' physical and mental well‐being (Ripp et al., 2020; Shechter et al., 2020). Their resilience could have been further compromised by physical distancing, isolation, and loss of social support; also, the risk of losing friends and relatives and drastic, often unsettling, changes in the ways of working (Lung et al., 2009).

It has been reported that when human‐to‐human transmission of COVID‐19 was officially confirmed, it caused public panic and distress (Qiu et al., 2020). Plus, Chew et al. (2020) underlined the association between the stress of the current pandemic in the people directly involved in the fight against the virus. A cross‐sectional study conducted in New York in April 2020 concluded that healthcare workers experienced substantial distress, including acute anxiety, depression symptoms, and sleep disturbances (Shechter et al., 2020). Similarly, a Pakistani (Salman et al., 2020) and a Nepali (Khanal et al., 2020) study reported that healthcare workers were experiencing considerable depression and anxiety. Also, the consequences of previous epidemics on healthcare workers, such as the severe acute respiratory syndrome (SARS) and Ebola, have been well documented (Liu et al., 2012).

During this pandemic, the world has seen many crises, including socio‐economic, educational, and psychological ones (Bodrud‐Doza et al., 2020; Daniel, 2020). Frontline healthcare workers have played a significant role by shielding and protecting the population, providing necessary healthcare, absorbing immense psychological pressures, and trying their best to deliver what was required. Even before it was officially declared a pandemic, healthcare workers had been putting their lives at risk, trying to tackle the situation and reducing the virus’ spread. All of it was an unbearable burden on their shoulders, one which they are still carrying. In the long run, their fight against the pandemic could prove costly, compromising their physical and psychological well‐being due to the emotional distress and sleep disturbances (Alimoradi et al., 2021b, 2021a; Tasnim et al., 2020).

In light of the continuing spread of the virus, and as fatigue adds up and the emotional burden becomes heavier, it is imperative to pay attention to the healthcare workers' mental health and well‐being (Lu et al., 2020). Huang and Zhao (2020) and Shanafelt et al. (2020) noted as early as February 2020 that the COVID‐19 outbreak has had significant consequences related, among other things, to sleep quality. A study from Bahrain reported that 60% of both frontline and non‐frontline healthcare workers had poor sleep quality combined with moderate‐to‐severe psychological stress (Jahrami et al., 2020). Such information is scarce in countries like Pakistan, India, and Nepal, and this study aimed to fill that gap. It is necessary to have a better picture of the present situation in these countries to make strategic use of resources already overloaded by the pandemic. It is for this reason that the present study set out to explore the impact of the COVID‐19 pandemic on sleep quality and the general mental health of healthcare workers in Pakistan, India, and Nepal.

## 2 MATERIALS AND METHODS

### 2.1 Participants and procedure

The present was a multi‐centric cross‐sectional, online survey‐based study, designed to explore the comparable descriptive estimates of sleep quality and psychological distress in healthcare workers from Pakistan, India, and Nepal. The study also examined the predictive relationship between sleep quality and psychological distress in the context of diverse personal and work‐related characteristics. Ethics approval was obtained from COMSATS University, Lahore, Pakistan. The present study targeted healthcare workers from India, Pakistan, and Nepal. Data collection used a convenient sampling technique. The inclusion criteria were (i) willingness to participate, (ii) being a healthcare worker, (iii) being a permanent resident of either India, Pakistan, or Nepal, and (iv) living in the country of origin since the outbreak of COVID‐19.

### 2.2 Measures

The online questionnaire consisted of three parts, namely, (i) socio‐demographics, (ii) General Health Questionnaire (GHQ‐12) (Goldberg & Williams, 1998), and (iii) Pittsburgh Sleep Quality Index (PSQI) (Buysse et al., 1989). The research team translated the English version of these two questionnaires into Hindi, Urdu, and Nepali.

#### 2.2.1 Socio‐demographics

These were questions about age, sex, marital status, whether there were children present at home (yes or no), country of residence, whether the person lived in a rural or urban area, level of healthcare services (primary, secondary, or tertiary) offered by the institution in which the participant worked, and whether the participant was actively working during the COVID‐19 pandemic. There were also two COVID‐19 related questions: (1) Do you have any COVID‐19 related symptoms? (yes or no), (2) Have you been diagnosed with COVID‐19 by a health professional? (yes or no).

#### 2.2.2 General Health Questionnaire

The GHQ‐12 consists of 12 items (Goldberg & Williams, 1998). These items assess the presence of current psychological distress using a 4‐point scale (from 0 to 3). Participants' responses generate a total score ranging from 0 to 36, with higher scores indicating worse health. Estimated alpha coefficients for GHQ‐12 were found as 0.89.

#### 2.2.3 Pittsburgh Sleep Quality Index

This is a self‐rated questionnaire that assesses sleep quality and disturbances over a 1‐month time interval (Buysse et al., 1989). It consists of 19 individual items generating seven “component” scores, namely, subjective sleep quality, sleep latency, sleep duration, habitual sleep efficiency, sleep disturbances, use of sleeping medication, and daytime dysfunction. The sum of scores for these seven components yields one global score. Its scoring is calculated for the seven components. The component scores then produce a total score (range 0 to 21); higher scores indicate worse sleep quality.

### 2.3 Statistical analysis

Data were analyzed via descriptive and inferential statistics using SPSS V.24. Descriptive analysis was used to estimate the demographic and the study's descriptive characteristics such as mean, standard deviation, frequencies, composition ratio, percentages, and compare group differences in sleep quality and psychological distress. Chi‐square test of association was used to evaluate the association between study characteristics (e.g., between groups and levels of sleep and psychological distress). It was also tested for normality parameters during data screening considering a *p*‐value of <.05 (two tailed). Binary logistic regression models were applied to assess the predictive association between different groups (e.g., gender, healthcare services, active working during corona pandemic, COVID‐19 symptoms, and diagnosis) and sleep quality and psychological distress.

## 3 RESULTS

A total of 1790 participants completed the online survey. Table 1 shows respondents' demographic characteristics and whether participants actively worked during COVID‐19 and reported symptoms or a positive diagnosis of COVID‐19, and the relation between these characteristics and sleep quality and psychological distress. Of the 1790 participants, 45% were male, and 55% were female, 810 (45%) were from Pakistan, 812 (45%) from India, and 168 (10%) from Nepal. Correlation analysis shows a significant positive link between being a female healthcare worker (*M* = 14.28*, SD* = 7.41 vs. *M *= 11.15, *SD* = 5.76), working at a tertiary setting, and with COVID‐19 symptoms, with psychological distress and poor sleep quality at a significant level (*p *< .0001 *and p *< .01 respectively). Other study variables, such as actively working during COVID‐19 (yes or no) and having been diagnosed with COVID‐19 (yes or no) were also linked positively and significantly with poor sleep quality (*p *< .01) (see Table 1).

**TABLE 1:** Descriptive characteristics of demographic and study variables

		Psychological distress		Sleep quality	
Measures	*f* (%)	*M (SD)*	*r*	*M (SD)*	*r*
Gender			.230***		.096**
Male	806 (45%)	11.15 (5.76)		5.99 (2.93)	
Female	984 (55%)	14.28 (7.41)		6.61 (3.35)	
Level of healthcare			.133***		.067**
Primary	359 (20%)	11.53 (7.06)		5.92 (3.33)	
Secondary	309 (17%)	11.68 (6.47)		6.28 (3.10)	
Tertiary	1122 (63%)	13.63 (6.85)		6.47 (3.15)	
Working actively during COVID‐19			.020		.115**
No	703 (39%)	12.81 (7.16)		6.02 (3.15)	
Yes	1087 (61%)	12.91 (6.72)		6.53 (3.19)	
COVID‐19 symptoms			.101***		.136***
No	1703 (95%)	12.74 (6.85)		6.23 (3.14)	
Yes	87 (5%)	15.55 (7.39)		8.27 (3.50)	
COVID‐19 diagnosis			.020		.081**
No	1705 (95%)	12.84 (6.91)		6.27 (3.17)	
Yes	85 (5%)	13.53 (6.57)		7.48 (3.34)	
Country					
Pakistan	810 (45%)	13.00 (7.27)		6.80 (3.36)	
India	812 (45%)	12.88 (6.83)		5.90 (3.00)	
Nepal	168 (10%)	12.22 (5.17)		6.12 (2.89)	

*Abbreviations*: *M*, mean; *SD*, standard deviations.

^*^ *p* < .05.

** *p* < .01.

*** *p* < .001.

Table 2 indicates a significant prevalence of poor sleep quality (*χ* ^2 ^= 14.62*, p *< .001) and psychological distress (*χ* ^2 ^= 9.981*, p *< .001) across all three regions. Of the 1790 participants, 57% reported poor sleep, and this ratio was most dominant for the Pakistani sample, with 63%, followed by the Nepali (55%) and Indian (53%) samples. Out of the total number of participants, 10% reported high psychological distress levels, with a comparatively high ratio of 22% found in the Pakistani sample compared to 9% and 4% for Indian and Nepali samples.

**TABLE 2:** Cross‐cultural comparison of prevalence of sleep quality and psychological distress

	Group estimates (*n* = 1790)				Individual estimates		
Sleep quality	Good sleepers *f (%)*	Bad sleepers *f (%)*	χ^2^	*k*	*N*	Good sleepers *f (%)*	Bad sleepers *f (%)*
Pakistan	232 (16)	388 (26)	14.62**	.031***	820	232 (37)	388 (63)
India	339 (23)	371 (25)			812	339 (48)	371 (52)
Nepal	75 (5)	92 (6)			168	75 (44)	92 (55)
Total	646 (43)	851 (57)					
Psychological distress	Group estimates (*n* = 1790)				Individual estimates		
	Low distress	High distress	χ	*k*	*N*	Low distress	High distress
Pakistan	713 (40)	97 (5)	9.981**	.022***	820	713 (88)	97 (22)
India	735 (41)	77 (4)			812	735 (91)	(9)
Nepal	161 (9)	7 (1)			168	161 (96)	7 (4)
Total	1609 (90)	181 (10)					

*p < .05.

**p < .01.

A significant association of gender with distress levels (low and high) was found for all samples, except for Nepali participants, where only 1% of males and females reported a high level of psychological distress. Working actively during COVID‐19 was seen as significantly associated with psychological distress (low and high) (*χ* ^2 ^= .969*, p *< .01) only for the Indian sample, where 4% of those actively working during COVID‐19 reported a high level of psychological distress. There was also a significant association of COVID‐19 symptoms with psychological distress levels only for the Nepali sample (*χ* ^2 ^= .861, *p *< .05). In the case of sleep quality, there was a significant association with gender (*χ* ^2 ^= 8.079, *p *< .01), the level of healthcare service (primary, secondary, or tertiary) offered by the institution in which the participant worked (*χ* ^2 ^= 9.843, *p *< .01), and COVID‐19 related symptoms (*χ* ^2 ^= 7.607, *p *< .01) only for the Pakistani sample (see Table 3).

**TABLE 3:** Sleep quality and psychological distress in healthcare professionals during COVID‐19 pandemic

	Overall (*N* = 1790)			Pakistan (*N* = 810)			India (*N* = 812)			Nepal (*N* = 168)		
Psychological distress	L‐DIS *f* (%)	H‐DIS *f* (%)	*χ^2^* (p)	L‐DIS *f* (%)	H‐DIS *f* (%)	*χ^2^* (p)	L‐DIS *f* (%)	H‐DIS *f* (%)	*χ^2^* (p)	L‐DIS *f* (%)	H‐DIS *f* (%)	*χ^2^* (p)
Gender												
Male	783 (41)	23 (2)	**39.253 (.0001)**	344 (43)	11 (1)	**23.053 (.0001)**	348 (43)	10 (1)	**14.631 (.0001)**	91 (54)	2 (1)	.492 (.483)
Female	881 (50)	103 (7)		398 (49)	57 (7)		411 (51)	43 (5)		72 (44)	3 (1)	
Health care services												
Primary	334 (19)	25 (1)	4.899 (.08)	162 (20)	14 (1)	4.643 (.098)	141 (17)	11 (1)	2.489 (.288)	31 (19)	0 (0)	3.569 (.168)
Secondary	296 (17)	13 (1)		138 (17)	6 (1)		118 (15)	4 (1)		40 (25)	3 (1)	
Tertiary	1034 (58)	88 (4)		442 (55)	48 (6)		500 (62)	38 (4)		92 (55)	2 (1)	
Working in pandemic												
No	647 (36)	56 (3)	1.519 (.218)	292 (36)	32 (4)	1.541 (.214)	313 (38)	22 (3)	**.002 (.969)**	42 (25)	2 (1)	.508 (.476)
Yes	1017 (57)	70 (4)		450 (56)	36 (4)		446 (55)	31 (4)		121 (73)	3 (1)	
Covid‐19 symptoms												
No	1588 (88)	115 (7)	**4.39 (.036)**	684 (85)	59 (7)	2.410 (.121)	742 (91)	51 (6)	.510 (.475)	162 (97)	5 (2)	**.031 (.861)**
Yes	76 (4)	11 (1)		58 (7)	9 (1)		17 (2)	2 (1)		1 (1)	0 (0)	
Covid‐19 diagnosis												
No	1583 (88)	122 (7)	.742 (.389)	681 (84)	64 (8)	.462 (.497)	741 (91)	53 (7)	1.285 (.257)	161 (97)	5 (2)	.062 (.803)
Yes	81 (4)	4 (1)		61 (7)	4 (1)		18 (2)	0 (0)		2(1)	0(0)	
Sleep quality	G‐SLP	B‐SLP		G‐SLP	B‐SLP		G‐SLP	B‐SLP		G‐SLP	B‐SLP	
	*f* (%)	*f* (%)	*χ^2^* (p)	*f* (%)	*f* (%)	*χ^2^* (p)	*f* (%)	*f* (%)	*χ^2^* (p)	*f* (%)	*f* (%)	*χ^2^* (p)
Gender												
Male	365 (20)	441 (24)	**7.891 (.005)**	151 (18)	201 (25)	**8.079 (.004)**	169 (21)	184 (23)	.662 (.416)	45 (25)	48 (29)	1.182 (.277)
Female	391 (22)	593 (34)		149 (18)	319 (39)		202 (25)	257 (31)		30 (18)	45 (27)	
Health care services												
Primary	176 (10)	183 (10)	**11.246 (.004)**	83 (10)	92 (11)	**9.843 (.007)**	76 (9)	73 (9)	1.709 (.425)	17 (10)	14 (8)	3.007 (.222)
Secondary	116 (7)	193 (11)		47 (6)	96 (12)		54 (7)	68 (8)		15 (9)	28 (17)	
Tertiary	454 (25)	668 (37)		170 (21)	332 (40)		241 (30)	300 (37)		43 (26)	51 (30)	
Working in pandemic												
No	305 (17)	398 (22)	1.115 (.291)	118 (14)	204 (25)	.133 (.715)	165 (20)	168 (21)	2.480 (.115)	22 (13)	22 (13)	.692 (.405)
Yes	441 (25)	646 (36)		182 (22)	316 (39)		206 (25)	273 (34)		53 (32)	71 (42)	
Covid‐19 symptoms												
No	727 (41)	976 (54)	**14.282 (.0001)**	286 (35)	451 (55)	**7.607 (.006)**	366 (45)	417 (51)	3.114 (.078)	75 (44)	92 (55)	.811 (.368)
Yes	19 (1)	68 (4)		14 (2)	69 (8)		5 (1)	24 (3)		0 (0)	1 (1)	
Covid‐19 diagnosis												
No	720 (40)	985 (55)	**4.495 (.034)**	283 (35)	455 (56)	3.493 (.062)	363 (45)	421 (52)	.024 (.876)	74 (44)	92 (54)	**.024 (.878)**
Yes	26 (2)	59 (3)		17 (2)	65 (7)		8 (1)	20 (2)		1 (1)	1 (1)	

*Abbreviations*: B‐SLP, bad sleepers; G‐SLP, good sleepers; H‐DIS, high distress; L‐DIS, low distress.

In the binary logistic regression analysis (see Table 4), professional healthcare workers who were male (OR = −.29, 95% CI: .617–.908, *p *< .01), working in primary health services (OR = −.312, 95% CI: .574–.933, *p *< .05), and with no symptoms of COVID‐19 (OR = −.887, 95% CI: .233–.727, *p* < .01) were less likely to have disturbed sleep. For psychological distress, healthcare workers who were females (OR = −.29, 95% CI: .617– .908, *p* < .01) and had symptoms of COVID‐19 (OR = −.29, 95% CI: .617– .908, *p *< .01) were less likely to have psychological distress; similar results were found for the Pakistani sample as well. On the other hand, when logistic regression was run for the Indian and Nepali samples, variant estimations were observed. Indian females were less likely to report symptoms of psychological distress (OR = −1.293, 95% CI: .135–.558, *p *< .0001). For Nepali participants, healthcare workers who were males (OR = −.267, 95% CI: .632–.928, *p *< .01) and with no symptoms of COVID‐19 (OR = −.169, 95% CI: .497–.437, *p *< .0001) were less likely to have poor sleep quality. Whereas, on the measure of psychological distress, females (OR = −.1.216, 95% CI: .204–.431, *p *< .0001) with no symptoms of COVID‐19 (OR = −.1.152, 95% CI: .168–.595, *p *< .01) were less likely to report psychological distress.

**TABLE 4:** Logistic regression of sleep quality and psychological distress

		Sleep quality				Psychological distress			
Measures		OR	AOD	95% CI (AOR)(LL‐UL)	*p*	OR	AOD	95% CI (AOR)(LL‐UL)	*p*
Total sample	Male ^Ref: Female^	−.290	.748	(.617–.908)	.003	−1.337	.263	(.165–.419)	.0001
	Primary health services ^Ref: Tertiary^	−312	.732	(.574–.933)	.012	.024	1.024	(.639–1.641)	.922
	Secondary health services ^Ref: Tertiary^	.211	1.235	(.948–‐1.610)	.118	−.510	.600	(.327–1.101)	.099
	No active working ^Ref: active working^	−.099	.906	(.743–1.104)	.328	.180	1.197	(.823–1.741)	.347
	No symptoms of Covid‐19 ^Ref: Symptoms of Covid‐19^	−.887	.412	(.233–.728)	.002	−1.070	.343	(.161–.729)	.005
	No diagnosis of Covid‐19 ^Ref: diagnosis of COVID‐19^	−.182	.834	(.489–1.421)	.504	.911	2.486	(.787–7.850)	.121
Pakistan	Male ^Ref: Female^	−.435	.647	(.480–.873)	.004	−1.407	.245	(.125–.479)	.0001
	Primary health services ^Ref: Tertiary^	−.456	.634	(.443–.908)	.013	−.011	.989	(.521–1.880)	.974
	Secondary health services ^Ref: Tertiary^	.241	1.273	(.844–1.920)	.250	−.649	.523	(.215–1.272)	.153
	No active working ^Ref: active working^	.051	1.052	(.776‐1.426)	.743	.274	1.315	(.786–2.201)	.298
	No symptoms of Covid‐19 ^Ref: Symptoms of Covid‐19^	−.707	.493	(.246–.987)	.046	−.902	.406	(.169–.973)	.043
	No diagnosis of Covid‐19 ^Ref: diagnosis of COVID‐19^	−.350	.705	(.360–1.379)	.307	.702	2.018	(.603–6.756)	.255
India	Male ^Ref: Female^	−.136	.872	(.657–1.158)	.345	−1.293	.274	(.135–.558)	.0001
	Primary health services ^Ref: Tertiary^	−.181	.834	(.578‐1.205)	.334	.146	1.157	(.569–2.354)	.688
	Secondary health services ^Ref: Tertiary^	.099	1.104	(.737–1.654)	.631	−.732	.481	(.166–1.392)	.177
	No active working ^Ref: active working^	−.222	.801	(.600–1.071)	.134	−.046	.955	(.535–1.705)	.875
	No symptoms of Covid‐19 ^Ref: Symptoms of Covid‐19^	−.875	.417	(.143–1.214)	.109	−1.136	.321	(.066–1.571)	.161
	No diagnosis of Covid‐19 ^Ref: diagnosis of COVID‐19^	.200	1.222	(.452–3.302)	.693	18.908	—	–	.998
Nepal	Male ^Ref: Female^	−.267	.766	(.632–.928)	.007	‐1.216	.296	(.204–.431)	.0001
	Primary health services ^Ref: Tertiary^	.118	1.125	(.999–1.267)	.061	.144	1.155	(.936–1.424)	.179
	Secondary health services ^Ref: Tertiary^	—	—	—	—	—	—	–	–
	No active working ^Ref: active working^	−.074	.929	(.763–1.131)	.462	.113	.316	(.811–1.545)	.492
	No symptoms of Covid‐19 ^Ref: Symptoms of Covid‐19^	−.881	.414	(.235–.731)	.002	−1.152	1.318	(.168–.595)	.0001
	No diagnosis of Covid‐19 ^Ref: diagnosis of COVID‐19^	−.169	.845	(.497–1.437)	.533	.276	.254	(.586–2.962)	.504

*Abbreviations*: AOD, adjusted odd ratio; CI, confidence intervals; LL, lower limit; OR, odd ratio; UL, upper limit.

## 4 DISCUSSION

The COVID‐19 pandemic has represented a significant health risk directly imposed on all healthcare workers. Unfortunately, knowledge is still scarce regarding the mental health impact of the current pandemic on healthcare workers. The present study explored the pandemic's impact on healthcare workers from India, Pakistan, and Nepal regarding two particularly relevant aspects: sleep quality and mental health.

Overall, the majority of the sample reported having poor sleep quality, and a significant number of participants reported high psychological distress. A cross‐cultural comparison found some differences between the different groups of participants, particularly regarding psychological distress. Of the total number of participants, 57% reported having poor sleep quality, and 10% reported high psychological distress. Poor sleep quality was reported by a majority of participants across all three regions whereas psychological distress was found at higher numbers in participants from Pakistan (22%) when compared with those from India (9%) and Nepal (4%).

As could be expected, the present study found a significant association between the presence of COVID‐19 symptoms in healthcare workers and higher levels of psychological distress and poor sleep quality. These results emphasize the need to provide further support to healthcare workers showing COVID‐19 related symptoms, as its psychological impact could have significant consequences both in the short and long term. The study also found a significant association between actively working during the COVID‐19 pandemic and poor sleep quality; however, there was no statistically significant association between actively working during the COVID‐19 pandemic and psychological distress. A study conducted by Ferini‐Strambi et al. (2020) found that healthcare workers' sleep quality was adversely affected by the pandemic. Similarly, Wilson et al. (2020) found a relatively low prevalence of stress among healthcare professionals actively working during the pandemic in India. But these results contrast with a recent study conducted by Lee et al. (2020) in China amongst anesthesiologists and nurses that found higher psychological distress associated with the pandemic. These contradictions could result from the subjective character of these outcomes, which, as discussed below, can be considered as one of the limitations of the present study.

The present study also found that healthcare workers from tertiary care institutions had higher distress levels than those working at primary and secondary tier settings. These higher levels could be due to tertiary care hospitals being at the forefront of hospital admissions and COVID‐19 procedural management during the pandemic. Lai et al. (2020) reported similar findings in their study, where participants working in a second‐tier hospital were more likely to report severe symptoms of depression, anxiety, and insomnia. Similar to the results reported by the study conducted in China by Liu et al. (2019), the present study found that almost 64% of healthcare workers from tertiary‐care hospitals reported poor sleep quality, with the number going up to 82.7% when including healthcare workers from both secondary and tertiary setups.

In the present study, female healthcare workers reported psychological distress at a higher ratio than their male counterparts, except in Nepali participants. No gender was significantly associated with psychological distress and poor sleep quality in Nepali healthcare workers. One previous Nepali study also reported no significant association of gender with psychological distress or poor sleep (Khanal et al., 2020). Still, considering the total number of participants, female healthcare workers were more likely to report psychological distress, whether at the lower or higher end of the spectrum. A similar study conducted in China found that the female participants were more prone to experiencing stress and developing post‐traumatic stress disorder due to the pandemic compared to their male counterparts (Qiu et al., 2020). Regarding sleep quality, a significant association was also found between female gender and poor sleep quality when considering the total number of participants. Studies have shown psychological distress to be a significant cause of poor‐quality sleep (Kim & Dimsdale, 2007). Thus, the higher association of psychological distress with female healthcare workers could contribute to the same group's higher association with poor sleep quality.

As a general estimate, being a male healthcare worker at a primary healthcare institution and not exhibiting any COVID‐19 related symptoms was associated with better sleep quality, while being a female healthcare worker and presenting COVID‐19 symptoms seems to be causing high psychological distress. From a country‐specific point of view, Pakistani participants seem to follow these patterns, while Indian females were less likely to demonstrate symptoms of psychological distress. In contrast, in Nepali participants, males without COVID‐19 symptoms had a lower chance of experiencing poor sleep, and females without COVID‐19 were shown to have lower distress rates. Overall, in a sample of almost equal proportions of male and female healthcare workers, we found that female healthcare workers actively providing healthcare during the pandemic, especially in tertiary care settings, were more prone to developing bad sleep or poorer health quality. Additionally, we also found that experiencing COVID‐19‐related symptoms added to the burden already carried by the same group of the studied population.

The present study was not without limitations. It was conducted specifically with healthcare workers, so its results may not be generalizable to other professions. This is further emphasized by the fact that the study used a convenience sampling method. Additionally, the transversal design of the study could prevent a complete and prospective view of the health status of healthcare workers with respect to the progress of the pandemic. Future research is recommended to increase the sample size and broaden the professions to other healthcare fields. It would also be essential to include other variables in the analysis, such as shift working and self‐efficacy with sleep quality.

## 5 CONCLUSION

In conclusion, there was an overall significant positive association between female gender, ascending level of healthcare services (primary less than secondary, secondary followed by tertiary) offered by the institution in which the participant worked, and the presence of COVID‐19 symptoms with psychological distress and sleep quality. The present study also found a significant positive association between being diagnosed with COVID‐19 and working actively during the pandemic with poor sleep quality. Still, a cross‐regional analysis found some differences between the samples. There was a positive association between the female gender and level of psychological distress in all samples except for Nepali participants, and active working during COVID‐19 was significantly associated with psychological distress for the Indian participants.

## CONFLICT OF INTEREST

The authors declare that they have no known competing financial interests or personal relationships that could have appeared to influence the work reported in this paper.

## AUTHOR CONTRIBUTIONS

All authors contributed equally to the paper. Hamza Rafique Khan, Irfan Ullah, and Muhammad Junaid Tahir conceived and designed the study. The other authors participated by formulating the final protocol, designing, and supervising the data collection and creating the final dataset. Hamza Rafique Khan, Farzana Ashraf, and Irfan Ullah did the data analysis and wrote the first draft of the paper. All authors participated in interpreting the data and developing further stages and the final version of the paper.

### PEER REVIEW

The peer review history for this article is available at https://publons.com/publon/10.1002/brb3.2383

## Data Availability

The data that support the findings of this study are available from the corresponding author upon reasonable request.
